# Stroke in sickle cell disease in association with bilateral absence of the internal carotid arteries. Case report

**DOI:** 10.1186/s12883-022-02702-5

**Published:** 2022-05-17

**Authors:** Ivana Markovic, Zoran Milenkovic, Bosanka Jocic-Jakubi, Amna Al Futaisi, Kakaria Anupam Kakaria, Yasser Walli

**Affiliations:** 1 IvanaMarkovic, Sultan Qaboos University Hospital, Radiology Department, Muscat, Oman; 2 General Hospital “Sava Surgery”, Kej 29 Decembar 2, Niš, 18000 Serbia; 3 Child Health Department, Sultan Qaboos University Hospital, Muscat, Oman; 4 Hospital, Child Health, Sultan Qaboos University Hospital, College of Medicine and Health Sciences, Muscat, Oman; 5 Department of Radiology and Molecular Imaging, Sultan Qaboos University Hospital, Muscat, Oman

**Keywords:** Sickle cell disease, Stroke, Seizure, Internal carotid artery, Agenesis

## Abstract

**Background:**

Congenital absence of the internal carotid artery (ICA) is a highly infrequent congenital incidence and occurs in less than 0.01% of the population; bilateral absence is exceedingly rare, diagnosed below 10% of the unilateral absence of the ICA. Sickle cell disease (SCD) is a serious disorder and carries a high risk of stroke.

**Case presentation:**

We present a five-year-old child with SCD who experienced an ischemic stroke episode with epileptic seizures. Neuroimaging revealed the agenesis of both ICAs. The frequency, embryology, and collateral pathway of the vascular anomaly as the clinical presentation, of this rare hematologic disease, are discussed.

**Conclusions:**

Sickle cell disease (SCD) carries a high risk of stroke. Congenital absence of ICA occurs in less than 0.01% of the population; bilateral absence is diagnosed below 10% of the unilateral absence of the ICA.

## Background

Sickle cell disease (SCD) is a hereditary disorder and carries a high risk of stroke, sometimes followed by seizures [1]. The incidence of focal or generalized seizures ranges from 6 to 12% [2, 3], and they are 2–3 times more common than in patients without SCD [4]. We presented a five-year-old boy with sickle cell anemia who experienced an ischemic stroke with seizures. Neuroimaging investigation revealed the absence of both internal carotid arteries (ICAs). Congenital absence of ICAs is rare, occurring in less than 0.01% of the population [5, 6]. The bilateral absence is highly infrequent, and it has been diagnosed in less than 10% of patients with unilateral ICA agenesis.

## Case presentation

**Fig. 1:**
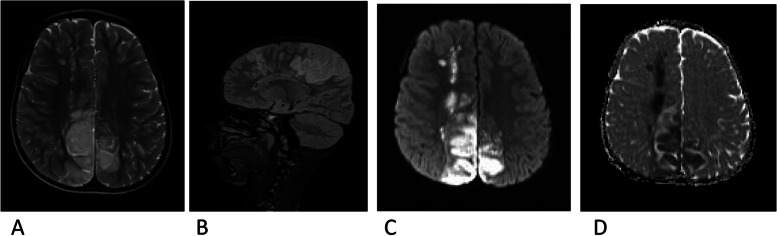
MR images axial T2W (**A**), sagittal FLAIR (**B**), axial DWI&ADC (**C**&**D**) revealed territories in the right frontal and both parietal lobes involved with acute watershed infarcts. Abbreviations: FLAIR- Fluid-attenuated inversion recovery, DWI diffusion-weighted imaging, ADC apparent diffusion coefficient map

**Fig. 2:**
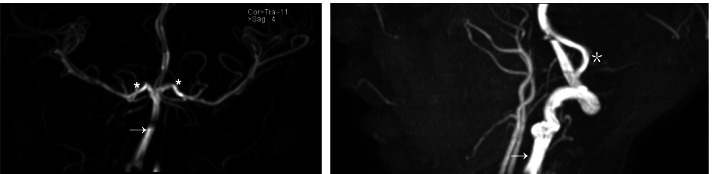
MRA-3D TOF showed no visible flow in both internal carotid arteries with reperfusion of anterior circulation through the prominent posterior circulation including basilar (arrows) and “posterior cerebral arteries” (asterisks).Abbreviations: TOF MRA - time of flight MR angiography

**Fig. 3:**
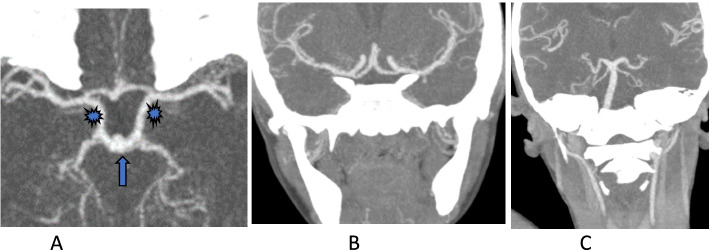
Brain arteries with corresponding almost equal segments look like “normal” circle of Willis; Anterior circulation is supplied through the prominent posterior circulation including dominant basilar (arrows) and posterior communicating arteries (asterisks) presenting on axial MIP CTA (A) and also on reformatting coronal MIP CTA images (B, C,). Abbreviations: CTA- Computed Tomography Angiography; MIP- maximum intensity projection

**Fig. 4:**
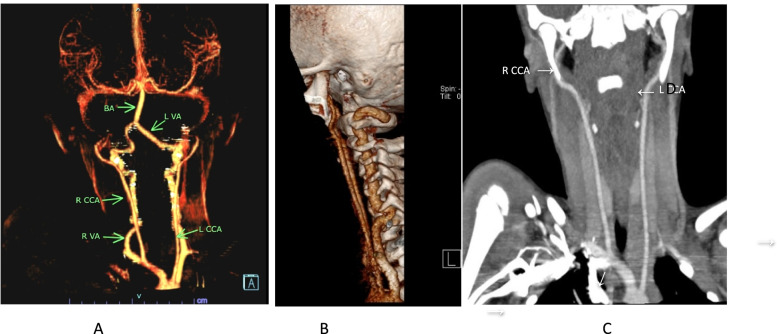
The complete absence of all segments of the internal carotid arteries on either side has shown in VR (**A**) and dominant vertebral arteries (**B**) while slim gracile common carotid arteries presented on coronal MIP CTA image (**C**)

**Fig. 5:**
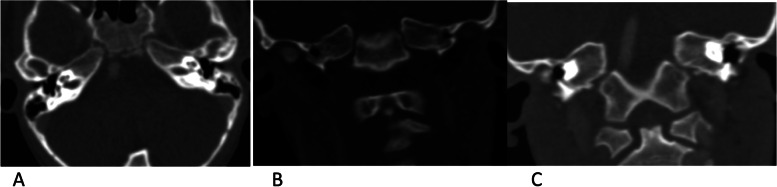
Absence of bony carotid canals in petrous bones detecting with bone window axial (**A**) and coronal (**B** and **C**) CT images, confirming the finding of agenesis both ICA

A five-year-old boy with a history of sickle cell disease was admitted to the hospital due to sudden left-side weakness, after an episode of severe abdominal pain and vomiting 8 days before admission. He also experienced a few attacks of a focal seizure characterized by rolling of the eye associated with facial twitching but the loss of consciousness, frothing and jerky movements were not observed. At the admission, the patient was crying, irritable, sick looking, but with no photophobia, neck stiffness or rigidity, no pallor, no jaundice. Laboratory findings: Hemoglobin 12.3; White blood cells (WBC) 10.3,plt; Neutrophil count 6.7. He received diazepam, exchange transfusion, and was referred to the teaching hospital in the capital city for further treatment and examination. In the referred hospital, the patient obtained three more transfusions and levetiracetam. The child recovered well in the next few days. The Magnetic resonance imaging (MRI) (MAGNETOM AERA 1.5 T SIEMENS) revealed acute watershed infarcts involving the right frontal and both parietal lobes (Fig. 1). Time of flight MR angiography (MRA-3D TOF) did not detect blood flow in both internal carotid arteries (Fig. 2). The additional evaluation continued with cerebral Computed tomography angiography (CTA) (SOMATOM SENSATION 64 slice SIEMENS) that showed established infarcts in corresponding arterial watershed territories of the middle cerebral artery and anterior cerebral artery. The origin of the large vessels of the neck from the aortic arch was of the standard size; however, common carotid arteries (CCA) were less than expected with no CCA bifurcation on either side. The absence of all segments of the internal carotid arteries (ICA) on both sides together with the absent bony carotid canals in the petrous bones confirmed the diagnosis of the agenesis of both internal carotid arteries. The anatomical parts of the circle of Willis had almost the same diameters and had no visible stenotic lesions on them (Figs. 3, 4 and 5).

On the day of discharge from the hospital, the child was conscious, understood verbal commands but was unable to carry verbal communication. He had spastic left side weakness (the leg is more involved than the arm); the gait was hemiparetic with circumduction.

He was undergoing regular transfusion therapy per month, and the antiepileptic medication (levetiracetam 270 mg BID stopped in October 2018 as he had not experienced a recurrence of seizure.

A month later, a neurological examination showed spastic left-sided hemiparesis with brisk, polykinetic reflexes, a permanent clonus, and a positive Babinski sign. The speech was preserved. A year later, spastic left hemiparesis is visible.

The patient is under the constant care of a hematologist (repeated blood transfusions for secondary prevention of stroke) and periodic checks by a neurologist (assessment of the patient’s neurological deficits and cognitive abilities). A new MRI / MRA is scheduled after 6 months (possible silent or open infarction and detection of new stenotic lesions in the main cerebral arteries).

Further monitoring was followed by certain difficulties. The patient lives in a rural area 15–20 km away from the local hospital. He gave up physical therapy because the father has no available time to take his son to therapeutic exercises every week. The patient did not show up for the scheduled MRI appointment.

The medical report 3 years later is as follows: The patient is on a regular transfusion, monitored by the hematology team. He is awake, alert, communicates well. There is spastic left hemiparesis with brisk deep tendon reflexes and Babinski’s sign. The gait is hemiparetic with circumduction. It has been suggested that the child receive ankle-foot orthosis. The attending doctor emphasized the importance of having physical therapy and an MRI.

The possibility of surgical revascularization has been considered and could be useful if the child experiences recurrent strokes (stroke or silent infarction) despite optimal blood transfusion therapy or episodes of TIA resistant to aspirin treatment.

## Discussion and conclusion

Congenital absence of ICA is a rare entity and occurs in less than 0.01% of the population [5, 6]. Bilateral absence of arteries is about 10% [7] and has been identified in 60 cases in the reviewed literature [8]. The absence may be related to agenesis or aplasia ICA [9–11]; however, agenesis indicates the complete failure of vessel formation and absence of carotid canal into the embryo what was the case of “our patient”. In aplasia, the carotid canal is reduced in size [12], the precursor is presented as a remnant, like a fibrous band [12, 13] and only one part of the artery may exist eg. ICA siphon [14].

Embryologically, the internal carotid artery originates from the cranial part of the dorsal aorta and the terminal segments of the primitive third aortic arch and fully develops by 6 weeks of gestation (fourth embryonic week) [15], while the carotid canal at the base of the skull determines size after the fifth or sixth week of fetal life [16, 17]. The anomaly requires the establishment of collateral flow to meet adequate brain vascularization. Usually, the circulation through the posterior communicating artery provides perfusion of the brain, forming the circle of Willis with its terminal branches, as is the case with the presented patient. Less common arterial supply comes from persistent embryonic vessels or external carotid artery collaterals.

Most of the patients with the absence of ICA are asymptomatic, especially in early childhood, as the collateral circulation satisfies brain perfusion [18].

Children with SCD tend to be affected by various forms of cerebrovascular complications, such as silent infarction, stroke, capillary sinus attack, venous thrombosis of the cavernous sinus, cerebral hemorrhage, and hyperviscosity syndrome (https://www.epilepsy.com/learn/professionals/co-existing-disorders/hematologic-disorders/sickle-cell-disease). Sickle cell anemia is an inherited hemoglobinopathy with abnormal red blood cells with mutated hemoglobin and reduced oxygen transport capacity [19]. Polymerization of hemoglobin molecules deforms erythrocytes, giving them a sickle shape. They are prone to hemolysis with secondary anemia. The event can lead to oxygen depletion in vital tissues and organs. The rigid, sticky sickle-shaped cells are less able to deform, may block small vessels, compromise blood flow, and “remain an influential model of microcirculatory pathology or” small vessel disease [20, 21]. In response to maintaining a stable supply of oxygen to the tissues, blood flow and the minute volume of the hearth increase, which makes individuals more susceptible to changes in blood perfusion and oxygen content in the artery.

The overall prevalence of stroke in sickle cell disease is 3.75% or 1.02 per 100 patients in children between 2 and 5 years of age [22]. Eleven percent of them experience stroke before the age of 20, or 0.5 to 1% per year suffered an insult over 20 years with a tendency for recurrence after 2–3 years [22, 23]. The risk of ischemic attack is 333 times higher than in healthy patients [24], or 250 to 400 times higher than the general population, and the probability of stroke up to the age of 40 is about 32% [19]. In a series of 448 SCD children with an average follow-up of 7.9 years, 30 (6.7%) patients suffered a stroke, and 11.5% of them experienced the event by 18 years of age [25]. Acute stroke and chronic cerebral ischemia in this disorder are serious conditions with long-term morbidity, physical disability, seizures, and cognitive impairment [26].

An ischemic stroke in SCD children dominates in the range between 54 and 75% and is highest during the first decade and after 30 years [22, 27, 28]. The predominant etiological factor is steno-occlusive arteriopathies, most often on the intracranial terminal segments of the internal carotid arteries, the proximal parts of the middle cerebral arteries, and the anterior cerebral arteries [27, 29–31]. Infrequently, steno-occlusive lesions are not the cause of overt stroke and silent cerebral infarction [29]. The Willis circle of ‘our patient’ did not demonstrate vasculopathy on the main intracranial arteries. Stotesbury et al. [30] proposed a model of neurological risk in SCD without visible vasculopathy: vasoocclusive factors such as hemoglobin polymerization, that causes hypercoagulability and endothelial adhesion of red blood cells, together with hemodynamic factors which provoke hemolysis and hemoglobin oxygen desaturation, reduce cerebrovascular dilatory reserve and extraction of exhausted oxygen. We could assume that our patient was in a state of increased flow derived blood only from the vertebrobasilar arterial system. Hypovolaemia that occurred during gastrointestinal pain and severe vomiting would be sufficient to cause a stroke.

Motor recovery in SCD is usually good, but cognitive impairment is almost inevitable due to white matter encompassment [32–34].

The patient experienced several focal motor seizures at the onset of stroke and was defined as acute seizures [35]. The child did not develop epilepsy in the further follow-up. The incidence of focal or generalized seizures ranges from 6 to 12% [2, 3]; a certain percentage of them will later develop epilepsy. Based on a cohort study of SCD it was reported that ‘epilepsy in persons with SCD is 2–3 times more common than in non-sickle populations’ [4]. In the Fox et al. [36] pediatric clinical materials about stroke in general with one-year follow-up, out of 109 patients who had seizures, 11 (10%) had active epilepsy. For each year younger, epilepsy was 20%. In a population-based study of Kaiser’s childhood stroke, an incidence rate of 3.6% per year was reported, with a cumulative incidence of 13% over 5 years [37].

Several studies have shown that blood transfusion is the therapy of choice for secondary prevention of stroke in SCD [38–40]**.** Therapy does not completely prevent the incidence of stroke and it is estimated that relapse ranges from ‘2.0 overt plus silent stroke / 100 patient-years in children with a history of silent stroke [35] ‘to 8, 1 overt plus silent strokes / 100 patient-years in those with previous open strokes ‘ [41]. Surgical revascularization may be useful in a limited, carefully screened group of children who have had recurrent strokes (a stroke or silent infarction) despite optimal blood transfusion therapy (suppressed hemoglobin C to < 30%) [42, 43] Hall et al. [35] performed revascularization surgery in 12 children with SCD and severe cerebral arteriopathy and managed to reduce the rate of open and silent infarction recurrence from 13.4 infarcts / 100 patient-years before revascularization to 0 infarcts / 100 patient-years after revascularization (*p* = 0.0057). So far no multicenter trial has been conducted to prove the effectiveness of the procedure [44].

The limitation of this presentation is the absence of catheter angiography that is superior to MRA and CTA for visualization of tertiary branches, cerebellar arteries [45], and possible moyamoya vessels. However, some medical centers do not have sufficient experience in handling this method in children as was the case in “our patient” [46].

## Conclusion

Sickle cell disease is a group of inherited red blood cell disorders and carries a high risk of stroke. Congenital absence of ICA is a rare inborn phenomenon and occurs in less than 0.01% of the population; bilateral absence is highly uncommon, diagnosed below 10% of the unilateral absence of the ICA. We presented a five-year-old boy with a history of sickle cell anemia who experienced an ischemic stroke episode with seizures. Neuroimaging investigation revealed the absence of both internal carotid arteries.

## Data Availability

All data containing relevant information to support the study findings are included in the manuscript.

## Abbreviations

SCD: Sickle cell disease
ICA: Internal carotid arteries
CCA: Common carotid arteries
BA: Basilar artery
VA: Vertebral artery
PCA: Posterior cerebral artery
MRI: Magnetic resonance imaging
MRA: Magnetic resonance angiography
CT: Computed tomography
CTA: Computed tomography angiography
VR: Volume rendering CT images
MIP: Maximum intensity projection
FLAIR: Fluid-attenuated inversion recovery
DWI: Diffusion-weighted imaging
ADC: Apparent diffusion coefficient map
TOF MRA: Time of flight MR angiography
